# Implementing social distancing policy measures in the battle against the coronavirus: protocol of a comparative study of Denmark and Sweden

**DOI:** 10.1186/s43058-020-00065-x

**Published:** 2020-09-21

**Authors:** Per Nilsen, Ida Seing, Carin Ericsson, Ove Andersen, Nina Thórný Stefánsdóttir, Tine Tjørnhøj-Thomsen, Thomas Kallemose, Jeanette Wassar Kirk

**Affiliations:** 1 Department of Health, Medical and Caring Sciences, Linköping University, Linköping, Sweden; 2 Department of Behavioural Sciences and Learning, Linköping University, Linköping, Sweden; 3 Cardiology and Speciality Medicine Centre, Region Östergötland, Linköping, Sweden; 4 Department of Clinical Research, Copenhagen University Hospital, Amager and Hvidovre, Hvidovre, Denmark; 5 Department of Health and Social Context, National Institute of Public Health, University of Southern Denmark, Copenhagen, Denmark; 6 Department of Public Health, Nursing, Aarhus University, Aarhus, Denmark

**Keywords:** Policy, Implementation, Social distancing, Evidence, Expert advice, Comparative research

## Abstract

**Background:**

Social distancing policies to ensure physical distance between people have become a crucial strategy in the battle against the spread of the coronavirus. The aim of this project is to analyze and compare social distancing policies implemented in Denmark and Sweden in 2020. Despite many similarities between the two countries, their response to the coronavirus pandemic differed markedly. Whereas authorities in Denmark initiated mandatory regulations and many severe restrictions, Swedish authorities predominantly promoted voluntary recommendations.

**Methods:**

The project is an interdisciplinary collaboration between researchers in Denmark and Sweden with different disciplinary backgrounds. The project is based on a comparative analysis, an approach that attempts to reach conclusions beyond single cases and to explain differences and similarities between objects of analysis and relations between objects against the backdrop of their contextual conditions. Data will be gathered by means of document analysis, qualitative interviews, and a questionnaire survey to address three research questions: (1) What social distancing policies regarding the coronavirus have been formulated and implemented, who are the policymakers behind the policy measures, which implementers are expected to implement the measures, and who are the targets that the measures ultimately seek to influence? (2) How have the social distancing policies and policy measures been justified, and what types of knowledge form the basis for the measures? and (3) What are the differences and similarities in citizens’ perceptions of acceptability and compliance with social distancing policy measures in relation to the coronavirus?

**Discussion:**

To create a structure for addressing the three research questions, the project applies a theoretical framework informed by the policy and implementation science literatures. The framework consists of five interdependent domains that have an impact on policy implementation: (1) policymakers, (2) policy characteristics, (3) implementers, (4) targets, and (5) policy environment. Details of the framework are provided in the article.

Contributions to the literature

- The paper describes a framework for analyzing policy implementation that merges knowledge from two fields by combining elements from policy research and implementation science.
- The project involves researchers from two countries and applies a comparative research approach to reach conclusions concerning implementation of social distancing policies beyond each country and to explain differences and similarities between the two countries, thus preventing over generalizations based on the researchers’ experiences and perceptions.
- The paper discusses the tension between relying on research evidence and expert advice in situations where research findings are limited and/or inconsistent and there is no “best practice”.

## Introduction

The global battle against the spread of SARS-CoV-2 (severe acute respiratory syndrome coronavirus 2), henceforth coronavirus, and coronavirus disease (COVID-19) requires extensive public cooperation and compliance with public health policies to be effective. Politicians and public health authorities in many countries have communicated to citizens about the urgency to comply with non-pharmaceutical (non-medical) requirements to slow the spread of the virus. A common approach globally has been social distancing to ensure physical distance between people. The rationale behind social distancing is that the virus spread will slow down by measures such as staying home, avoiding crowds, and refraining from touching one another to diminish transmission of virus [1]. Social distancing policies have become a crucial part of mitigating pandemic influenza globally [2].

This project analyzes and compares social distancing policies implemented in Denmark and Sweden in 2020 to curb the spread of coronavirus. Both Nordic countries developed and implemented numerous policies for social distancing. Governments in both countries worked with medical and epidemiologic experts in public health authorities nationally and internationally to select policy measures to achieve the policy objectives regarding social distancing. The two neighboring countries share many cultural, historical, political, and economic characteristics. Both countries have a publicly funded health and welfare system, and both have a constitutional monarchy, with limited power for the ruler; power is exercised through governments and ministers. Sweden has nearly twice the population of Denmark (10 million versus 5.8 million) but is more sparsely populated (24 inhabitants/km^2^ versus 132 inhabitants/km^2^) [3]. However, despite many similarities in the two countries, their response to the coronavirus crisis differed markedly. Whereas authorities in Denmark initiated mandatory regulations and many severe restrictions, Swedish authorities predominantly promoted voluntary recommendations.

Denmark implemented regulations that mandated a lockdown of the country’s borders and the closing of schools, restaurants, and shopping malls [2]. Compared with many other European countries, Denmark was an early mover. A number of restrictions were in place early on in the pandemic, including limiting gatherings to ten people, recommending the workforce to stay home, and closing the borders [4]. Denmark’s response was broadly similar to many other European countries, but the lockdown was less restrictive than those in France or the UK, for example. Thus, there was no stay-at-home order, and many shops remained open although bars, gyms, and hairdressers were closed [5]. Internationally, Denmark was referred to as something of a “test case” in its swift early response of mandatory measures [6].

In contrast, Sweden’s response to tackling the coronavirus focused on voluntary recommendations [7]. This approach was justified with reference to the lack of evidence for many of the regulatory measures undertaken elsewhere, e.g., school closures [7]. Media and academic scholars attributed the Swedish approach to the citizens’ high trust in public institutions, because this was argued to create favorable conditions for voluntary state-led recommendations [8]. Further, voluntary measures for improved public health have a long tradition in Sweden [9]. The Swedish response took into account the broader societal and economic consequences of social distancing measures. For example, it was stressed how closing down schools would mean losing an estimated 25% of the workforce (i.e., parents would need to stay home with their children), including many health care workers [10]. The ability to continue with the measures over a longer time period was also emphasized as an important factor [11].

Internationally, Sweden’s response attracted a considerable amount of media attention, being labeled a “relaxed” or “light touch” approach. The high COVID-19 death rate per capita in an international perspective (despite the challenges of comparing figures between countries) raised concern about the effectiveness of the approach [12].

### Social distancing: definition, effectiveness, costs, and acceptance

Previous pandemics provide clues about what forms of social distancing might be relevant for slowing down the spread of COVID-19. Most evidence comes from other viral respiratory illnesses that can spread by particles remaining in the air after an infected individual coughs or sneezes, such as influenza, which has caused a number of pandemics in the twentieth and twenty-first centuries, including the Spanish flu in 1918–1920 and the less extensive but more recent H1N1flu pandemic in 2009–2010. Unlike COVID-19, Ebola is not transmitted through the air but through direct physical contact, although the Ebola outbreak in West Africa in 2014–2015 also offered lessons on social distancing. In general, research on pandemics has shown that it is difficult to contain influenza geographically in the location where they emerge, and international spread is difficult to avoid for more than a short period [13].

Social distancing is usually defined as the practice of maintaining a greater than usual physical distance from other people or avoiding direct contact with people or objects in public places to minimize exposure and reduce the transmission of an infection [7, 8, 14]. The Centers for Disease Control and Prevention (CDC) define social distancing as practices for reducing the frequency and closeness of contact between people in order to decrease the risk of transmission of disease [15]. Although the term social distancing continues to be used, the World Health Organization initiated the use of “physical distancing” in spring 2020 because it more accurately reflects the practices involved given that digital technology has enabled people to be socially connected without being physically in the same room or space with other people [16].

We have identified three systematic reviews that provide information on the effectiveness of social distancing measures to reduce virus transmission, the social and economic costs of such measures, and acceptance of the measures among the general public: Fong et al. [17], Mahtani et al. [13], and Rashid et al. [18]. Knowledge on social distancing has been obtained from clinical and epidemiologic studies, studies based on mathematical modeling of virus spread, as well as through personal clinical experiences about the impact of social distancing measures.

The three reviews have arrived at similar conclusions. Rashid et al. [18] stated that the overall quality of the evidence is not strong and that most social distancing measures were found to be moderately effective. Similarly, Mahtani et al. [13] said that “although limited, the best available evidence appears to support social distancing measures as a means of reducing transmission and delaying spread” and concluded that “staggered and cumulative implementation of these interventions may prove most effective.” They emphasized that the timing and duration of such measures were critical. Fong et al. [17] concluded that social distancing measures could be effective interventions to reduce transmission and mitigate the impact of an influenza pandemic, but “the evidence base for these measures was derived largely from observational studies and simulation studies; thus, the overall quality of evidence is relatively low.”

School closure has been found to be moderately effective in reducing the transmission of influenza and in delaying the peak of an epidemic [19–24]. However, this measure has been associated with very high economic costs and negative social impacts although this largely depends on the duration of the closure [25].

With regard to workplace-related interventions, the evidence available is rather limited. Interventions such as work closure and working from home have been found to be modestly effective, although they are usually considered to be acceptable, particularly if compensation is provided [25, 26]. Research suggests that a fairly high proportion of workplace closures are required for such a measure to have significant impact. However, workplace closures could cause considerable economic difficulties and widespread social distress [25, 27].

Working from home has been found to be potentially moderately effective in reducing the transmission of influenza [25]. Although the social and economic costs associated with working from home are likely to be moderate compared with business closures, they would have a disproportionate effect on small businesses and self-employed people [28].

With regard to voluntary self-isolation of individuals, this has been found to be moderately effective. There is an increased risk of intrahousehold transmission, particularly where bathroom facilities are shared [29–31]. The costs of voluntary isolation have not been investigated in any depth, but they are thought to be moderate and relate primarily to employment loss as a result of having to stay home from work [28]. Findings concerning the acceptability of voluntary self-isolation are somewhat variable [32–34].

The effects of cancelling mass gatherings depend on numerous factors such as event duration, degree of crowding, type of venue, and event timing in relation to the period either side of the peak of the epidemic [35]. There is some evidence that it is possible to safely organize a mass gathering in the midst of pandemic influenza by taking rigorous control measures [36]. The public’s acceptability of cancellation of mass gatherings is likely to vary depending on the characteristics of the gathering [37].

### Research needs

The literature review suggests that existing research findings on social distancing are inconsistent and that the quality of the accumulated evidence is not overly strong. Overall, there is rather limited evidence of the effectiveness for social distancing measures; many measures are described in the research literature as being “moderately effective.” Findings also tend to be inconsistent regarding the acceptance as well as social and economic costs of social distancing measures. Different interpretations of the social distancing research findings likely influence the development and implementation of policies and use of policy measures to prevent the spread of the coronavirus.

The approaches used by Denmark and Sweden offer a unique opportunity to study the development, implementation, and compliance concerning social distancing policies and policy measures. Knowledge about the more typical and common approach taken by Denmark and the more unusual approach adopted by Sweden is required to understand and explain how and why different social distancing measures may work or not. We have not been able to find any studies focusing on Denmark or Sweden or comparative research concerning social distancing policy measures.

The paucity of knowledge underscores the relevance of investigating what social distancing policies and measures have been implemented and the characteristics of these as well as differences and similarities between Denmark and Sweden. It is highly relevant to investigate how governments and public health authorities argue and provide reasons for policy regulations and recommendations and the extent to which the measures are, or are proclaimed to be, based on research- or experience-based knowledge as well as the importance of political “saleability” and ideological considerations regarding what measures are taken. There is also a need to investigate how measures are perceived, accepted (or not), and acted upon and complied with by the public and by different groups of citizens in society.

### Aims and research questions

The aim of this project is to generate new knowledge concerning important aspects of policies and policy measures for social distancing in Denmark and Sweden to prevent the spread of the coronavirus. Both countries have developed and implemented numerous policies and introduced many policy measures concerning social distancing, but their responses to the crisis differ markedly.

The following research questions will be investigated with regard to Denmark and Sweden:

1. What social distancing policies regarding the coronavirus have been formulated and implemented in 2020, who are the policymakers behind the policy measures, who are expected to implement the measures, and who do the measures ultimately seek to influence?
2. How have the social distancing policies and policy measures been justified and what types of knowledge form the basis for the measures concerning the coronavirus?
3. What are the differences and similarities in citizens’ perceptions of acceptability of and compliance with social distancing policy measures in relation to the coronavirus?

### Theoretical framework

#### Key definitions

Although definitions of policies are multifarious, public policies can be defined as objectives formulated and/or actions taken by a government (e.g., regarding social distancing) to address a societal problem (e.g., regarding the spread of the coronavirus). A policy may sometimes be identifiable in terms of a decision but often involves a series of decisions or what may be seen as more of a general orientation. Moreover, policies tend to change over time [38]. This project concerns policies that are regulatory, which means that they specify conditions and constraints for individual and collective behavior [39, 40] to achieve social distancing.

Policy characteristics refer to attributes of the formulated regulatory policy (i.e., the “implementation object”), such as the clarity of the policy objectives or the policy’s justification with regard to the perceived need it is intended to address [41]. Policymakers are those formally responsible for setting the agenda, articulating policies, and selecting policy measures [38].

Policy measures are the “something” that is done to realize the objective of a policy. Thus, policy measures are the more concrete, specific actions (interventions, initiatives, etc.) carried out to implement a policy [38]. Measures are also referred to as policy instruments or policy tools [42]. The policy literature distinguishes between different categories of measures, e.g., enforcement, education, and engineering (modifying the environment) measures for improved traffic safety [43].

Implementers of public policies are typically organizations, such as governmental authorities and public and private entities. The implementers are the “link” between the policymakers and the intended targets (see below) of the policies, ensuring that the policy measures are implemented as planned. The bottom-up policy implementation perspective has emphasized the relevance of understanding the influence of the implementers [44]. Lipsky [45] showed the importance of decisional latitude of street-level bureaucrats, suggesting that the influence of new knowledge must be considered alongside the implementers’ long-standing practices. Contemporary perspectives on policy research usually take a holistic view of implementers and describe complex networks of stakeholders such as individuals, organizations, and inter-organizational relations, thus making it difficult to determine who the implementers are [13].

Targets of policies are the individuals or organizations whose behaviors a policy ultimately seeks to influence through legislation, sanctions, regulations, provision of information, and other policy measures. Targets in policy research are also referred to as clients or recipients [44].

The policy environment is the context in which a policy is developed and implemented, incorporating, for example, historic, political, cultural, and resource contexts [46]. The policy environment is also referred to as the setting, conditions, and structure [41]. This environment represents forces that are, at least partially, beyond the control of the policymakers, implementers, and targets [44]. Bottom-up policy implementation researchers have focused a great deal of attention on the context of implementation [47].

Two types of results of policy implementation processes are typically distinguished: output is the impact on the implementers and outcome is the impact on the targets, e.g., citizens and organizations. Outputs are generally administrative decisions such as decisions to fund larger numbers of teachers, psychologists, or police officers, whereas comparable outcomes may include improved student assessments, reduced mental health problems, and lower crime rates in society [41]. Outcomes can often be difficult to attribute directly to outputs [44]. The project will not study the results quantitatively, but various aspects relating to both outputs and outcomes will be addressed in the interview and survey questionnaire studies because they likely influence the development and implementation of policies and use of various measures.

#### The policy cycle

**Fig. 1:**
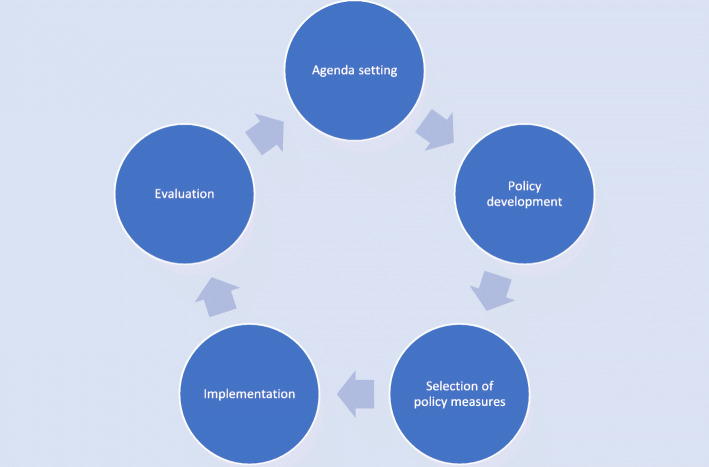
The policy cycle

The study of public policymaking has traditionally applied a policy cycle model that describes a number of activities of the policy process (Fig. 1): (1) agenda setting, i.e., identifying the objectives of a policy; (2) developing a policy to achieve those objectives; (3) selection of policy measures to realize the policy; (4) implementation of the policy and policy measures; and (5) evaluation of the policy and measures [42]. Thus, a policy cycle divides the policy process into a series of stages, from a notional starting point at which policymakers begin to think about a policy problem to a notional end point at which policy measures have been implemented and policymakers consider how successful they have been. Although the use of the cycle model for policy studies has diminished, the activities are still relevant [41].

In this project, the agenda setting (stage 1) is predetermined, i.e., the objectives related to preventing the spread of the coronavirus by means of social distancing. The focus of the project is on the implementation of policies and policy measures (stage 4), but the success of this process is influenced by aspects related to the policy development (stage 2) and what policy measures have been selected (stage 3). The project applies a framework that recognizes the dynamic interdependency of these activities (see below). The project does not investigate whether or how the policies have been evaluated by the policymakers (stage 5), but the research itself may provide information of relevance for such an undertaking.

#### Policy implementation factors

Policy research has described numerous conditions for successfully implementing policies and policy measures. For example, the importance of clear and well-communicated policy objectives has been emphasized [41, 47]. The policy must be a good solution to the problem [48], and the required resources must be committed to implement the policy [47]. Further, policies need to be implemented by skillful implementers, e.g., public officials [42]. Policy researchers have categorized various conditions into frameworks that describe different types of influences, i.e., change factors, on policy implementation results. Frameworks range from comprehensive checklists of large numbers of specific factors to frameworks describing a limited number of overarching factors of relevance for explaining policy implementation success (or failure) [41, 44].

**Fig. 2:**
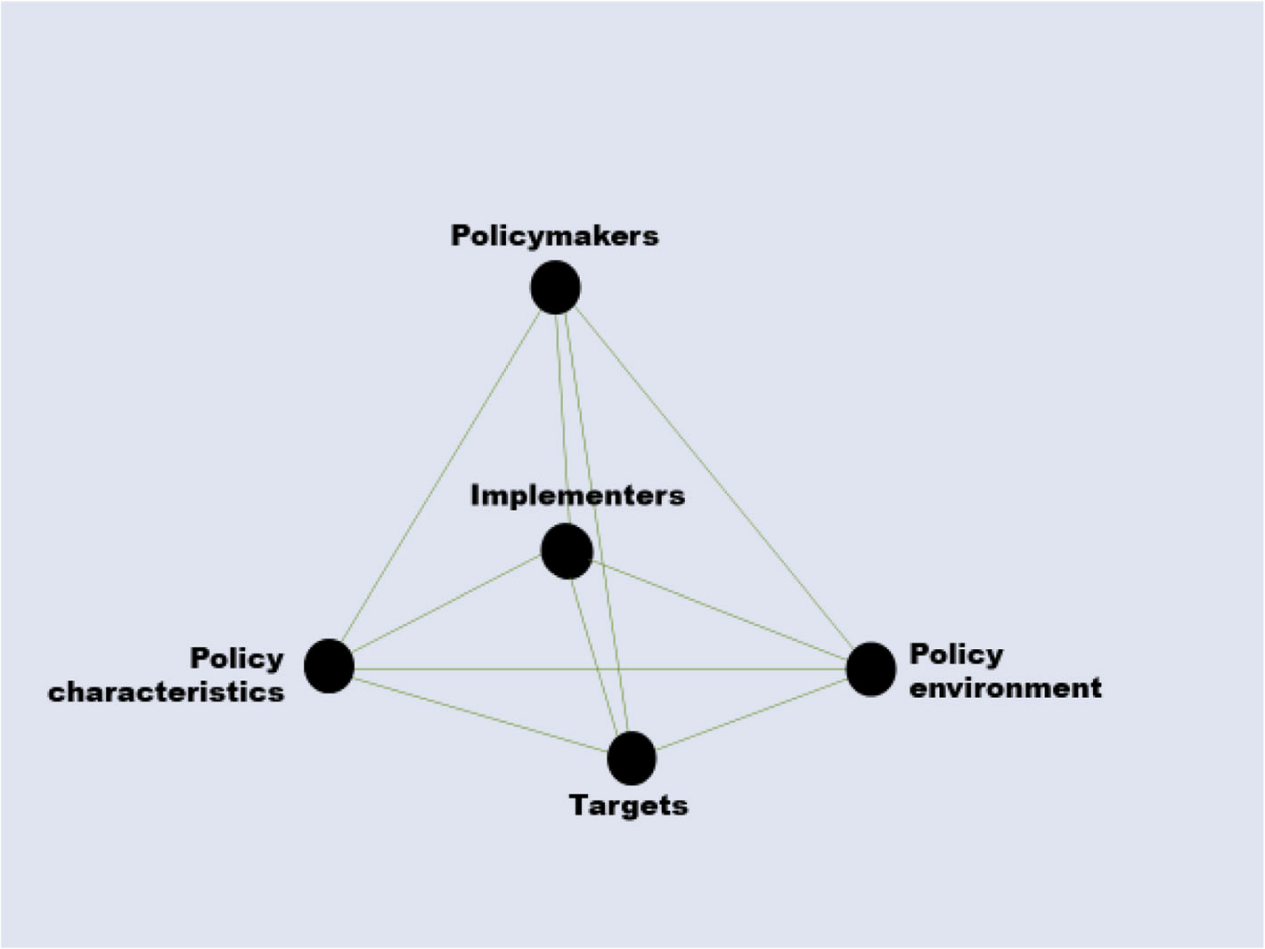
Influences on policy implementation

To create a structure for addressing the three research questions, the project uses a theoretical framework informed by the policy literature (e.g., [33, 36, 38, 39, 49]) and implementation science [50, 51]. The framework consists of five interdependent domains that have an impact on policy implementation: (1) policymakers; (2) policy characteristics; (3) implementers; (4) targets; and (5) policy environment. The domains are illustrated in Fig. 2, with lines connecting all domains with each other to reflect the inherent interdependency.

The five domains account for aspects related to three stages of the policy cycle: the policy development (stage 2), selected policy measures (stage 3), and the implementation of these measures (stage 4). The domains are interdependent, underscoring the relevance of understanding policies in holistic terms because their success or failure depends on combinations of different factors [50].

## Methods

### Design

The project is an interdisciplinary collaboration between researchers in the two countries with different disciplinary backgrounds, representing both social science and medical science. The research project is based on a comparative analysis [52] and uses both quantitative and qualitative methods to address the three research questions. Data will be gathered by means of document analysis, interviews, and a questionnaire survey.

### Applying the theoretical framework

The five interdependent domains of policy implementation factors will be applied to address the three research questions. The domains will be operationalized as follows:

- *Policymakers*: the formal decision makers at the governmental level as well as decision makers in the relevant public health authorities in both countries.
- *Policy characteristics*: the attributes of the formulated social distancing policies and policy measures, e.g., the extent to which the policy measures are mandated by law with sanctions (i.e., regulations) or voluntary recommendations, in both countries.
- *Implementers*: organizations in both countries that are responsible for carrying out the policy measures and those whose purpose is to ensure that the measures are complied with.
- *Targets*: citizens in Denmark and Sweden whose behaviors the social distancing policies ultimately seek to influence.
- *Policy environment*: includes social, political, cultural, and population characteristics in Denmark and Sweden as well as international and national media channels, economic actors, and knowledge producers (e.g., the research community and the World Health Organization) that exert pressure on policymakers and may have an impact on the development, implementation, and results of policies in the two countries.

### Data collection for research question 1

Research question 1 will be addressed by means of document analysis [53]. The data consist of different forms of formal public policies, articulated in text documents by government offices and public health authorities in both countries. The document data will be analyzed using qualitative content analysis [54].

### Data collection for research question 2

Research question 2 will be addressed using document analysis [53] to examine all social distancing policy measures undertaken in Denmark and Sweden to determine how the policies and policy measures have been justified and what types of knowledge form the basis for the measures.

Research question 2 will also be investigated using semi-structured interviews with policymakers (e.g., politicians, higher public officials/experts in authorities and government offices) who have had a central role in policymaking processes in the two countries. We estimate that about 20 interviews in each country will be sufficient to capture key aspects of the research question.

A purposive sample of informants will be recruited by means of identification of individuals in the document analysis and through snowballing [49]. A purposive sample is based on the researcher’s knowledge about the study and the population, the main goal being to focus on particular characteristics of a population, i.e., policymakers, that are of interest and which will best enable us to answer the research questions. A heterogeneous sample will be sought to produce a diverse range of cases relevant to the phenomenon being examined, thus providing as much insight as possible [49]. The data will be analyzed using thematic analysis [54].

### Data collection for research question 3

Research question 3 will be addressed with a cross-sectional questionnaire survey, which will include questions about acceptability and social distancing behavior.

The Swedish survey will use recruitment based on a web panel administered by a company specializing in survey research. The web panel in Sweden consists of a sample of individuals who are representative of the age, sex, and region of residence of the population aged 18–64 years. In Denmark, data will be obtained from Statistics Denmark and recruitment will be administered from the Department of Clinical Research.

No sample size calculation will be performed because the exploratory design aims to investigate each question in the survey. No differences found between the groups will be considered conclusive; they will only be hypothesis generating with regard to which areas of the implementation might be of interest for further study.

Survey responses will be analyzed and presented as mean/median with standard deviation/interquartile range of continuous variables and frequencies with percentages for categorical variables. Comparison of survey questions between Denmark and Sweden will be done pairwise by t test (or Wilcoxon rank sum test if distribution is not normal) for continuous variables and by chi-squared test for categorical variables.

Research question 3 will also be addressed through semi-structured interviews. About 30 semi-structured telephone interviews will be conducted in both countries with citizens from the three population groups: young people (15–25 years), families with children (< 10 years), and older people (65+ years). Thematic analysis will be used to analyze the interview data [54].

### Ethical considerations

The project will adhere to the directives of the Helsinki Declaration [55]. Ethical approval will be sought from the Danish Data Protection Agency and the system of Health Research Ethics Committees in Denmark and Sweden. Written informed consent will be obtained from all participants taking part in the research conducted as part of the project. It will be made clear to participants that they can withdraw from the research at any time if they choose to do so. All interviews will be conducted in undisturbed locations. Anonymity will be ascertained by assigning a code to each participant in the field notes and interviews.

The researchers are aware of potential ethical issues regarding sensitive questions concerning individuals’ perceptions and experiences concerning the coronavirus and policy measures taken in the fight against the spread. In the interviews, the researchers are aware of power issues; an interview is not a conversation between two equal individuals. Measures will be taken to ensure confidentiality of the participants. The project team will provide contact information so that the participants can contact any of the researchers should questions or comments arise during or after participating in the interviews.

Data in paper form will be stored in a fire-proof, locked safe at Hvidovre University Hospital. Electronic data such as audio files and transcripts will be stored in a folder with restricted entry located on a drive set up by the IT department of the Capital Region of Denmark. This is in compliance with ethical principles to ascertain anonymity of the participants. The new EU General Data Protection Regulation will be followed to keep identities confidential and the data secure. Any information that might enable identification will be removed.

## Discussion

The research project addresses an under-researched issue of high importance and strategic relevance in the fight against the spread of the coronavirus and the handling of other future pandemics. The aim is to generate new knowledge concerning important aspects of policies and policy measures for social distancing developed and implemented in Denmark and Sweden to prevent the spread of the coronavirus. The project accounts for the perspectives of policymakers, implementers, and citizens to provide a full, in-depth understanding of a complex phenomenon.

The project is based on a comparative analysis of social distancing policies and measures in Denmark and Sweden. Despite similar political systems, Denmark and Sweden have taken different approaches to social distancing, making the comparison of various aspects of the responses of the two countries highly relevant. Comparative research is usually understood as the contrast among different macro-level units, such as world regions, countries, sub-national regions, and social milieus at one point or more points in time. Comparative research attempts to reach conclusions beyond single cases and explains differences and similarities between objects of analysis and relations between objects against the backdrop of their contextual conditions. Comparative analysis can provide understanding of one’s own society by placing its familiar characteristics against those of other systems. Comparison heightens the awareness of other patterns of thinking and acting, thereby casting new light on one’s own arrangements. This type of research can prevent scholars from over-generalizing based on their own experiences and perceptions [52].

The project has significant importance in light of the inconsistent research findings concerning the effectiveness, costs, and acceptance of social distancing. Currently, there exists no specific “best practice” for social distancing measures that can be relied upon. This limitation makes it difficult to promote social distancing policies or measures as “evidence-based,” which has become increasingly expected of public policies [41]. Evidence-based (or “science-based”) policymaking uses the best available research to guide decisions at all stages of the policy process. Evidence-based policymaking is aimed at reducing wasteful spending, expanding effective interventions and programs, and strengthening accountability [56]. The project will explore the relevance of the lack of evidence-based social distancing policymaking for policymakers, implementers, and citizens in the two countries, including the citizens’ acceptance of and compliance with the policy measures.

The inconsistent research findings concerning social distancing measures likely makes the policymakers in the two countries more inclined to seek policy advice from experts and expert authorities. There are high and increasing expectations for an evidence-based practice in areas such as health care and social work, but there is a similar movement in the political field [41, 57]. The increased involvement of experts in policymaking has been termed “expertilization” [58], which is believed to signal a rise of “expertocracy” [59], “epistocracy” [60], or “epistemocracy” [61]. This development has been depicted in both positive and critical lights, as both a democratization of expertise and a threat to democracy [62, 63]. The project will explore the uncertainty regarding the evidence base for social distancing in terms of its influence on the policymakers, implementers, and citizens, including the role of expert involvement in the policy process and the relationship and potential tension between politics and expertise with regard to social distancing policymaking.

In summary, the research project is expected to produce new knowledge of relevance for the study of policy processes and implementation. Based on a theoretical framework informed by research from the policy literature and implementation science, the project will explore the importance and interaction of various factors on the development, implementation, and compliance concerning public policies and policy measures. The project will also generate knowledge about issues of considerable importance for policymaking regarding the present and future policies and measures taken to prevent the spread of the coronavirus as well as other future viruses. The project has the potential to identify effective ways to facilitate the acceptance of and compliance with policy measures regarding social distancing, thus generating implementable knowledge in the battle against the coronavirus and future viruses. The outcomes of the project will benefit several types of recipients in Denmark and Sweden who will obtain knowledge on the different approaches on social distancing measures chosen by Denmark and Sweden.

## Data Availability

The data will be available from the corresponding author on reasonable request.
